# Tumor Selectivity of Oncolytic Parvoviruses: From *in vitro* and Animal Models to Cancer Patients

**DOI:** 10.3389/fbioe.2015.00055

**Published:** 2015-04-22

**Authors:** Assia L. Angelova, Karsten Geletneky, Jürg P. F. Nüesch, Jean Rommelaere

**Affiliations:** ^1^Infection and Cancer Program, Division of Tumor Virology, German Cancer Research Center (DKFZ), Heidelberg, Germany; ^2^Department of Neurosurgery, University of Heidelberg, Heidelberg, Germany

**Keywords:** oncolytic virotherapy of cancer, oncolytic viruses, oncotropism, oncoselectivity, parvovirus H-1, preclinical assessment, toxicological studies, safety profile

## Abstract

*Oncolytic virotherapy* of cancer is among the innovative modalities being under development and especially promising for targeting tumors, which are resistant to conventional treatments. Presently, at least a dozen of viruses, belonging to nine different virus families, are being tested within the frames of various clinical studies in cancer patients. Continuously growing preclinical evidence showing that the autonomous rat parvovirus H-1 (H-1PV) is able to kill tumor cells that resist conventional treatments and to achieve a complete cure of various human tumors in animal models argues for its inclusion in the arsenal of oncolytic viruses with an especially promising bench to bedside translation potential. Oncolytic parvovirus safe administration to humans relies on the intrinsic preference of these agents for quickly proliferating, metabolically, and biochemically disturbed tumor versus normal cells (*tumor selectivity* or *oncotropism*). The present review summarizes and discusses (i) preclinical evidence of H-1PV innocuousness for normal cells and healthy tissues *in vitro* and in animals, respectively, (ii) toxicological assessments of H-1PV mono- or combined therapy in tumor-bearing virus-permissive animal models, as well as (iii) historical results of experimental infection of human cancer patients with H-1PV. Altogether, these data argue against a risk of H-1PV inducing significant toxic effects in human patients. This highly favorable *safety profile* allowed the translation of H-1PV preclinical research into a Phase I/IIa clinical trial being currently in progress.

## The Concept of Oncolytic Virotherapy for Cancer Treatment

Oncolytic virotherapy is one of the innovative modalities under development to target tumors that are refractory to conventional surgical and radio/chemotherapeutic treatments. While the possibility of using some viruses to fight cancer was put forward at the beginning of the twentieth century, the field underwent striking revival in the last three decades, along with the development of molecular virology and genetic engineering. The term “oncolytic viruses” (OVs) designates non-pathogenic live viruses that can infect and kill malignant cells without causing any harm to normal tissues. Because of space constraints, the concept of oncolytic virotherapy will be only briefly outlined below, and citations will be limited to some recent review articles (Liu et al., 2007; Haseley et al., 2009; Meerani and Yao, 2010; Wong et al., 2010; Friedman et al., 2012; Russel et al., 2012; Singh et al., 2012; Ahmed, 2013; Bartlett et al., 2013; Goldufsky et al., 2013; Vacchelli et al., 2013; Lichty et al., 2014; Vähä-Koskela and Hinkkanen, 2014; Woller et al., 2014), to which readers are referred for further details.

**Figure 1:**
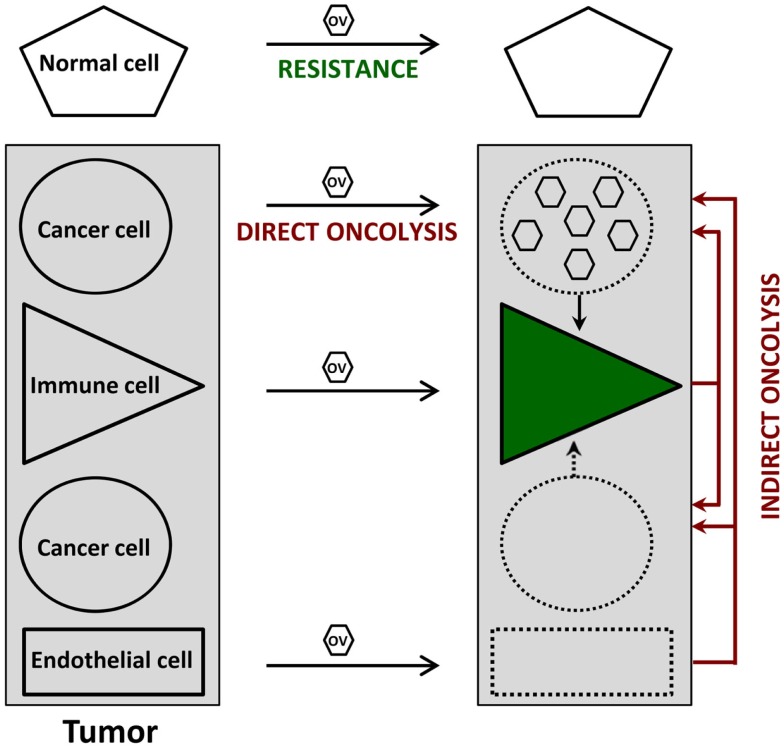
**Direct and indirect anti-tumor effects of oncolytic viruses (OVs)**. Normal cells resist OV infection because of viral life-cycle blockage prior to the induction of cytopathic effects. In contrast, OVs can disturb at least three cell types within the tumor. Infected cancer cells undergo an immunogenic type of death (direct oncolysis), which is sometimes (e.g., figure) but not always accompanied by virus production; this oncolysis leads to the activation of various immune cells (green), irrespective of their direct virus infection, thereby priming anti-cancer immune responses. Furthermore, the (abortive) OV infection of immune and endothelial cells inside the tumor results in cytotoxic cytokine production and anti-vascular/angiogenic effects, respectively, which both contribute to virus-mediated oncolysis in an indirect way. Uninfected tumor cells can also serve as targets for these indirect oncolytic effects.

Cancer virotherapy is based on four main properties of OVs, as depicted in Figure 1.

(i) *Oncoselectivity* is an obvious *sine qua non-condition* for oncolytic virotherapy. The selectivity of OV infection and replication for tumor cells is an inherent feature of certain virus species, or a result of virus adaptation or targeted genetic engineering. This *oncotropism* reflects the dependence of distinct step(s) of the OV life-cycle on tumor-specific molecular (epi)genetic alterations. It results in particular from the fact that, on the one hand, most tumors have evolved mechanisms to suppress responses used by normal cells to limit virus infection, and on the other hand, signaling pathways promoting the growth of tumor cells favor virus replication as well.
(ii) *Oncolysis* can be induced in a *direct way* by OVs as a result of virus replication and/or expression of viral cytotoxic gene products in infected tumor cells. A major asset of OVs lies in their multimechanistic mode of malignant cell killing, which differs from the cell death processes triggered by conventional anti-cancer agents. This peculiarity can be rationalized by the virus need to prevent premature cytopathic effects (CPE) from interfering with virus production, and is therapeutically exploited to overcome the resistance developed by many tumor cells to conventional therapies.
(iii) *Indirect bystander oncolytic effects* also contribute to a significant extent to the oncosuppressive activity of OVs, which can mediate in this way the killing of uninfected cancer cells. These indirect OV-induced oncolytic effects can result from tumor vasculature disruption and angiogenesis, release of toxic cytokines from infected tumor-resident or infiltrating immune cells, and even more importantly, from systemic anti-tumor immune responses. Indeed, the tumor cell death caused by OVs is often immunogenic, leading to the activation of innate immune cells and to efficient presentation of tumor-associated antigens eliciting adaptive anti-tumor immunity. Oncolytic virotherapy can be considered from either virocentric or immunocentric points of view, depending on whether emphasis is placed on direct virus-induced oncolysis or on virus-mediated stimulation of anti-tumor immune responses. In animal models, both mechanisms appear to act in combination, while having different relative weights depending on the individual target tumor.
(iv) *Replication-competent OVs* are used in the hope of achieving intratumoral amplification of the initial inoculum and production of progeny virions, which can spread to non-infected tumor cells, including distant metastases, until the entire tumor tissue is affected. While this multiplication has been observed in some animal tumor models, extensive OV spread at sites of tumor growth remains to be documented in clinical settings.

The potential of the oncolytic virotherapeutic approach is supported by substantial evidence of OV-induced oncosuppressive effects at both preclinical and clinical levels. More than a thousand patients have now been treated with OVs by intratumoral injection and/or intravenous infusion during Phase I–III clinical trials. Compelling evidence of OV-induced anti-cancer immunity was obtained for a recombinant oncolytic herpes simplex virus by showing that its intratumoral administration to patients with metastatic malignant melanoma led to the complete regression of injected and uninjected tumors in 8/50 treated patients (Kaufman et al., 2010). It has also been shown that an oncolytic vaccinia virus can reach tumor sites by extravasating from tumor blood vessels (Breitbach et al., 2013), lending credit to the use of systemic OV administration for metastases targeting. Indeed, in an earlier clinical trial, the authors have shown that a vaccinia vaccine-derived oncolytic poxvirus used as a vehicle for delivery and expression of transgenes, achieves cancer-selective replication after intravenous infusion in patients with advanced, treatment-refractory solid tumors (Breitbach et al., 2011). OV spread at tumor sites following systemic administration has been clinically demonstrated with yet another virus. An oncolytic reovirus was intravenously infused before surgery to resect colorectal cancer liver metastases. Reovirus immune cell carriage and delivery as well as recovery of replicating virus from the tumor was achieved, confirming intravenous reovirus targeting of metastatic colon carcinoma (Adair et al., 2012). The OVs, which are most advanced in clinical developments, include oncolytic herpes simplex, vaccinia, reo-, and adenoviruses, with the latter being licensed in China for use against head and neck cancer (Garber, 2006). The pace of clinical activities in the field has accelerated considerably, and OVs belonging to not less than nine different virus families are presently the subject of various clinical studies in cancer patients.

These clinical studies confirmed that OVs, as expected from their oncoselectivity, can be safely administered to humans. Indeed, the clinical tolerability of OVs has overall been excellent, and dose-limiting toxicities were only rarely observed. Interestingly, the most common adverse effects – transient flu-like symptoms – do not overlap with those caused by other anti-cancer agents, supporting the possibility of combining oncolytic virotherapy with current therapeutic modalities. Since future trials will likely use higher OV doses, it is not guaranteed that effective virotherapy will always be devoid of toxicity. However, this concern has to be balanced against the risk of current therapies, which approach the upper limit of tolerability. One OV-specific safety risk relates to viral spread from the treated patient to contacts. However, OV transmission has not been observed so far, although limited virus shedding in bodily fluids has occasionally been reported (Makower et al., 2003; Pecora and Lorence, 2007; Hughes et al., 2014).

Among OVs, the autonomous rat parvovirus H-1, the subject of this review, deserves special consideration as candidate anti-cancer agent. Continuously growing preclinical evidence demonstrates the ability of this virus to kill tumor cells that resist conventional anti-cancer treatments and to achieve a complete cure of various tumors in animal models, importantly, while being innocuous for non-transformed cells and normal tissues (Rommelaere et al., 2010). Currently, parvovirus H-1 is in a Phase I/IIa clinical trial designed to document its maximum tolerated dose and safety profile in patients with recurrent glioblastoma multiforme.

## The Oncolytic H-1 Parvovirus

**Figure 2:**
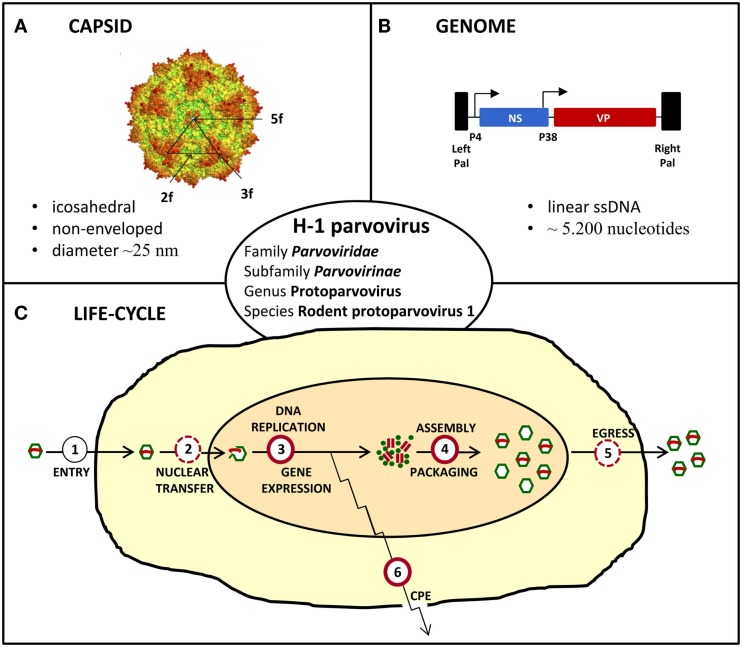
**Characteristics of H-1 parvovirus**. **(A)** *In silico* model of H-1PV capsid surface showing the two, three, and fivefold axes of symmetry (Allaume et al., 2012). **(B)** Simplified viral gene expression map. The viral single-stranded (ss) DNA genome ends in unique palindromic sequences (Pal), which serve as self-priming origins of replication for the synthesis of double-stranded replication forms/transcription templates. Transcription is controlled by the P4 and P38 promoters that are indicated by arrows and direct expression of non-structural (NS) and capsid (VP) proteins, respectively. **(C)** Simplified scheme of the viral life-cycle depicting the main steps leading to virus production and induction of cytopathic effects (CPE). Known and putative oncogenic transformation-stimulated steps are indicated by full and dashed red circles, respectively.

The oncolytic H-1 parvovirus (H-1PV) belongs to the family *Parvoviridae*, subfamily *Parvovirinae*, genus Protoparvovirus, and species Rodent protoparvovirus 1 (Figure 2). Like all parvoviruses, H-1PV is a small (25 nm in diameter) non-enveloped virus characterized by an icosahedral capsid and a linear single-stranded DNA genome of approximately 5,000 nucleotides (Tattersall, 2006). Viral genomic DNA comprises two transcription units that are controlled by the P4 and P38 promoters and code for the non-structural (NS) and structural (VP) viral proteins, respectively (Cotmore and Tattersall, 2014; Cotmore et al., 2014). The NS protein NS1 plays a major role in the viral life-cycle, by being essential for viral DNA replication, gene expression, and virus-induced cytotoxic effects (Nüesch, 2006; Hristov et al., 2010; Nüesch et al., 2012; Li et al., 2013). H-1PV replicates autonomously in target cells, in close dependence on cellular proliferation and differentiation factors (Cornelis et al., 2006). The natural host of H-1PV is the rat, infection of whom can either be pathogenic and even lethal (in immunologically unprotected fetuses and neonates) or clinically inapparent (in adult animals). As documented below, under *in vitro* conditions, the virus preferentially replicates in and kills transformed or tumor-derived rat and human cell cultures, without inducing cytotoxicity in their corresponding non-transformed/non-malignant counterparts (Rommelaere et al., 2005, 2010). Moreover, the above observations illustrative of parvovirus H-1 *oncoselectivity* have been further extended to animal models, in which H-1PV was reported to efficiently suppress tumor formation and to cause striking regression of established tumors (*oncosuppression*, see below) (Rommelaere and Cornelis, 1991).

## Mechanisms of Rodent Protoparvovirus Oncoselectivity

Parvovirus intrinsic oncoselectivity is a complex phenomenon based in part on multiple tumor cell-specific determinants, which are under-represented in non-malignant cells. The availability of cellular replication and transcription factors, the overexpression of cellular proteins known to interact with parvoviral ones (with NS1, in particular), the activation of metabolic pathways involved in the functional regulation of NS1, are all contributing to parvovirus preference for tumor, and not for normal cells. The molecular pathways involved in H-1PV tumor cell targeting are overviewed in a recent publication from our laboratory (Nüesch et al., 2012), while the most important *in vitro* and animal studies providing evidence of parvovirus oncoselectivity and innocuousness in a non-malignant environment are summarized in the present review (see following sections). Although it is feasible to attenuate PVs by genetic engineering (Daeffler et al., 2003), the use of these viruses as anti-cancer agents is not based on this strategy since the innocuousness of wild-type PVs to normal tissues avoids the need to attenuate undesirable toxicity.

To our knowledge, there is no evidence to indicate that PV preference for infecting tumor cells results from a higher competence of these cells for virus binding and internalization. Instead, neoplastic cells appear to provide an intracellular milieu that is especially permissive for at least part or the full course of the PV life-cycle. This higher permissiveness of tumor cells cannot be traced back to a single factor, but rather involves multiple cellular factors that control different steps of the PV life-cycle and each give infection a distinct boost (Figure 2). These factors are thus likely to cooperate in promoting virus infection, with the impact of later-acting factors depending on the completion of earlier steps in the viral cycle. A number of tumor cells provide all the factors that are necessary for full virus replication, resulting in progeny particle production and cell lysis. Some other tumor cells are semi-permissive (and get killed in the absence of progeny virions release), and a few remain resistant to infection. It should also be stated that man is not the natural host of rodent PVs, and normal human cells may pose different and/or additional restrictions to PV infection, compared to rodent cells. This can be exemplified by our recent work showing that the PDK1/PKC/PKB cascade – which regulates the functioning of the replicative and cytotoxic PV protein NS1 – is induced by the mouse parvovirus MVM in mouse but not in human cells (Lachmann et al., 2008; Bär et al., 2015). In consequence, the constitutive activation of this cascade through an unconventional mechanism in human tumor cells represents a human-specific determinant of the oncoselectivity of PVs.

**Figure 3:**
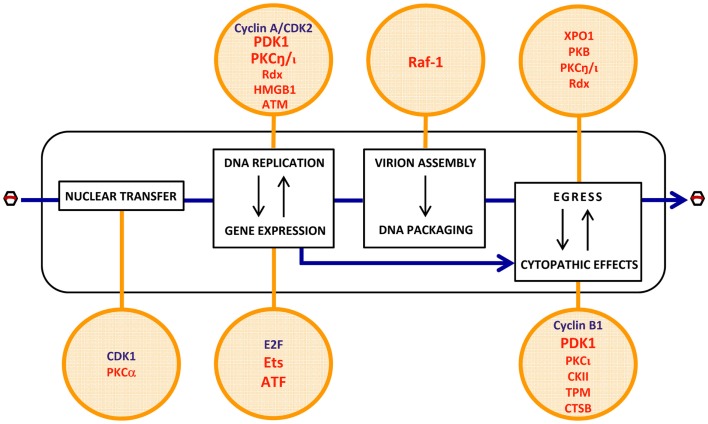
**Putative cell determinants of PV oncoselectivity**. Indicated steps of the PV life-cycle (rectangles) were shown to be controlled by cellular factors (circles) known to be regulated at gene amplification, expression, and functional levels by cell proliferation (blue) and oncogenic transformation (red). The list of factors is not exhaustive and exemplifies candidate mediators of the enhanced permissiveness of neoplastic cells for PV infection. Evidence of the contribution of these factors to PV oncotropism is experimental for a few of them (PDK1, PKCη, Ets, ATF, Raf-1) but circumstantial for the others. For more details, see main text (pp. 4–5). CDK2, cyclin-dependent kinase 2; PDK1, phosphoinositide-dependent kinase 1; PKC, protein kinase C; Rdx, radixin; HMGB1, high-mobility group box protein 1; ATM, ataxia-telangiectasia mutated protein; Raf-1, rapidly accelerated fibrosarcoma-1 protein; XPO1, exportin-1; PKB, protein kinase B; E2F, transcription factor E2F; Ets, E26 transformation-specific transcription factor; ATF, activating transcription factor; CKII, casein kinase II; TPM, tropomyosin; CTSB, cathepsin B.

Most of the cellular determinants of PV oncoselectivity, as identified so far, control the viral life-cycle in a positive way (Figure 3).

- Some of these factors are not strictly tumor-specific but characteristic of proliferating cells. Examples thereof are cyclin A/CDK2 (Bashir et al., 2000; Adeyemi and Pintel, 2012) and E_2_F (Deleu et al., 1999), which are cellular S-phase markers and control the conversion of the PV single-stranded DNA genome into double-stranded replicative forms/transcription templates, and the activation of the PV early promoter P4, respectively. Both factors are thus essential for the onset of PV replication, contributing to the S-phase dependency of these viruses. Although a fraction of tumor cells can be dormant and some normal tissues are rapidly self-renewing, the proliferating component of many tumors distinguishes them from essentially quiescent surrounding normal tissues, offering a target for PV infection and thereby contributing to the enhancement of virus replication and CPE in neoplastic versus normal tissues.
- Other determinants of PV oncoselectivity are more specific for malignantly transformed cells in which they are overexpressed or activated as a result of (epi)genetic alterations occurring in these cells. A few factors of this type have been shown to promote distinct steps of the PV life-cycle. These steps include:

(i) *viral entry*: PKCα- and CDK1-dependent nuclear envelope breakdown (Porwal et al., 2013).
(ii) *viral gene expression*: activation of the early PV promoter P4 by Ets and ATF transcription factors (Perros et al., 1995; Fuks et al., 1996).
(iii) *viral DNA amplification*: activation of the PV replicative protein NS1 through PDK1, PKC, and radixin (Rdx)-mediated phosphorylation (Dettwiler et al., 1999; Lachmann et al., 2003, 2008; Nüesch et al., 2009; Bär et al., 2015); HMGB1-dependent initiation of DNA amplification from the right-hand viral origin (Cotmore and Tattersall, 1998; Cotmore et al., 2000); facilitation of viral replication through an ATM kinase-mediated DNA damage response (Adeyemi et al., 2010).
(iv) *viral progeny capsid assembly*: Raf-1 phosphorylation-dependent nuclear transport of capsid intermediates (Riolobos et al., 2010).
(v) *virions maturation*: XPO1, PKB, PKC, and Rdx-dependent egress (Eichwald et al., 2002; Nüesch et al., 2009; Bär et al., 2013, 2015).
(vi) *cytopathic effects*: expression of potential PV targets, including distinct tropomyosin (TPM) isoforms (Nüesch and Rommelaere, 2007), or checkpoint factors, e.g., cyclin B1 (Adeyemi and Pintel, 2014); production of cytotoxic effectors, such as cathepsin B (CTSB) (Di Piazza et al., 2007) and CKII (Nüesch and Rommelaere, 2006); PDK1 and PKCι-dependent activation of NS1 cytotoxic function (Nüesch and Rommelaere, 2006; Bär et al., 2015).

The role played by some of these factors in the oncotropism of PVs was supported by the fact that their helper function was stimulated in transformed cells, rescuing at least to some extent the viral life-cycle defect in normal cells.

The oncoselectivity of a number of OVs can also be traced back to the frequent deficiency, in tumor cells, of mechanisms that allow normal cells to counteract virus infection, in particular, the type I interferon and stress responses. Along this line, the mouse Protoparvovirus MVM was shown to both induce the type I interferon response and be sensitive to it in normal mouse cells, while a still elusive evasion mechanism appeared to be triggered by the virus in their transformed counterparts, preventing interferons from being produced (Grekova et al., 2010a,b). It follows that in this system, PV oncoselectivity consisted of an additional component, namely, the absence of negative modulators of PV infection in transformed cells. It is unclear whether these data can be extrapolated to human neoplastic versus normal cells. Various human tumor cells were recently reported to fail to develop a type I interferon response upon PV (including H-1PV) infection. However, this failure was also observed in normal human cells (Paglino et al., 2014), questioning whether the interferon response plays any role in the oncoselectivity of rodent protoparvoviruses in human cells. It should be again recalled in this regard that man is not the natural host of rodent protoparvoviruses, and some of the factors limiting the viral life-cycle may therefore differ between cells from natural and heterologous hosts.

## *In vitro* Evidence of H-1PV Oncoselectivity

The pioneering observations demonstrating that H-1PV fails to induce CPE in normal human (e.g., embryonic kidney and amnion) cultures were published by Toolan and Ledinko (1965). H-1PV innocuousness for non-malignant cells has been later confirmed in a number of *in vitro* studies, as summarized in Table 1.

**Table 1:** ***In vitro* evidence of H-1PV oncoselectivity**.

Transformed/tumor cells (Tu)	Normal cells (N)	Tu versus N sensitization to H-1PV infection	Reference
Spontaneously transformed human amnion	Normal human amnion^a^	CPE in Tu No toxicity in N	Toolan and Ledinko (1965)
SV40-transformed human (fore)skin fibroblasts	Normal human (fore)skin fibroblasts^a^	Efficient Tu killing: active DNA and protein synthesis; abundant NS1 phosphorylation; productive Tu infection Minor cytotoxicity, no killing and abortive N infection	Chen et al. (1986, 1989), Faisst et al. (1989), Dupressoir et al. (1989), Van Pachterbeke et al. (1993, 1997), and Muharram et al. (2010)
Squamous cell carcinoma-derived human keratinocytes	Normal human breast skin keratinocytes^a^		
Breast carcinoma-derived human epithelial cells	Normal human mammary gland epithelial cells^a^		
Hepatoma-derived human cells	Normal human hepatocytes^a^	Tu killing through apoptosis No NS1 expression and no N killing	Moehler et al. (2001)
Glioma-derived human cells	Normal human astrocytes and glia^a^	Cathepsin-mediated Tu death Low NS1 levels and no N killing	Di Piazza et al. (2007) and Lacroix et al. (2010, 2014)
EBV-transformed and lymphoma/leukemia-derived human immune cells	Normal human and rat immune cells^a^	Efficient Tu killing and productive Tu infection Retained N viability and abortive N infection	Moehler et al. (2003), Angelova et al. (2009a), Grekova et al. (2010a,b), Moralès et al. (2012), and Raykov et al. (2013)

*^a^Non-established (low-passage-number) cultures*.

*CPE, cytopathic effect; EBV, Epstein–Barr virus*.

For example, H-1PV infection was quantitatively compared in a series of non-permanent cultures of *normal human (fore)skin fibroblasts* and their respective SV40-transformed counterparts. Although the virus was adsorbed and taken up by both normal and transformed cells, only in the latter, H-1PV was able to induce killing and progressive culture degeneration. In addition, a striking difference in the capacity of normal versus transformed fibroblasts to support viral DNA and protein synthesis was found. Only the SV40-transformants, but not their non-transformed counterparts, could provide the intracellular environment required to support the completion of H-1PV lytic cycle and the release of infectious progeny virions (Chen et al., 1986; Faisst et al., 1989). Similar observations were made when established keratinocyte cell lines derived from human squamous cell carcinoma were used. Efficient virus-induced killing and productive infection were seen in human tongue, cheek, supraglottis, and face squamous cell carcinoma-derived keratinocytes. In contrast, *normal human epidermal cells* isolated from healthy adult breast and used after one passage *in vitro* (low-passage-number cultures) were resistant to H-1PV infection (Chen et al., 1989; Faisst et al., 1989). In another study, established lines or low-passage-number *in vitro* cultures derived from either human breast tumor specimens or *normal peritumoral breast tissue* from the same patient were used to evidence H-1PV oncoselectivity. The striking oncolytic virus-induced cytotoxic effects observed in tumor-derived cultures were absent in the respective normal tissue-derived controls (Dupressoir et al., 1989; Van Pachterbeke et al., 1993, 1997). Furthermore, in invasive breast carcinoma-derived cells, in contrast to normal breast epithelium, a distinct phosphorylation pattern of the major viral cytotoxic protein NS1 was documented (Muharram et al., 2010). Selective H-1PV-induced killing of human liver carcinoma cells versus normal hepatocytes was also reported. Low-passage number cultures established from *healthy liver tissue* failed to support NS1 expression and were therefore refractory to lytic infection (Moehler et al., 2001). In order to prepare a preclinical platform for H-1PV clinical application in brain tumor patients and patients with other nervous system-originating tumors, virus toxicity was evaluated in *normal primary human infant astrocytes*, *glial cells*, and *cortical neurons* as well. The data showed that the morphology, metabolic activity, and membrane integrity of the above cells remained unaltered even after high-dose H-1PV infection. Whereas in malignant neuroblastoma cells, abundant NS1 expression was observed and found to result in cellular G2 arrest, no or only low NS1 levels could be detected in normal astrocytes and mixed glial cultures. Indeed, although the latter could take a recombinant EGFP-transducing virus up, transduction efficiency was more than 10-fold lower than in the neuroblastoma cell line reported to be the least sensitive to H-1PV-induced cytotoxicity (Lacroix et al., 2010, 2014). In agreement with these data, virus innocuousness for *normal human adult astrocytes* was also described. It was shown that while triggering lysosomal membrane permeabilization and cathepsin-mediated death in human glioma-derived cells, H-1PV was non-toxic for normal astrocyte cultures (Di Piazza et al., 2007). Experimental data illustrating absence of H-1PV-induced toxicity in normal cells came also from studies using preclinical non-Hodgkin lymphoma models. In contrast to Burkitt lymphoma-derived cells, which were highly susceptible to H-1PV-induced killing and supported high levels of progeny virions production, *non-malignant B lymphocytes*, including normal memory B cells, were shown to resist infection, with minor toxicity being observed only when cultures were infected with high H-1PV doses. Since normal B lymphocytes were unable to express EGFP after transduction with a recombinant EGFP-transducing virus, this minimal toxic effect was unrelated to viral gene expression (Angelova et al., 2009a). *Normal human immune cell* resistance to PV infection was reported by other authors as well. Immature or mature dendritic cells and monocytes were shown to retain viability after H-1PV challenge (Moehler et al., 2003), while, in contrast, the human histiocytic lymphoma-derived monocyte cell line U937 is among the *in vitro* systems being the most sensitive to H-1PV lytic effects (Rayet et al., 1998). Proofs of PV innocuousness for *normal peripheral blood mononuclear cells* are of particular importance in view of current and future H-1PV clinical applications. It was shown that although basal levels of viral DNA replicative forms were detected in the latter cells, no productive infection, even after mitogen stimulation, could be observed (Grekova et al., 2010a,b). Similar results were reported by Moralès et al. (2012) and Raykov et al. (2013).

## *In vivo* Evidence of H-1PV Oncoselectivity

After the *in vitro* experiments using different primary and low-passage-number cultures demonstrated that H-1PV infection of normal human cells is abortive, does not result in cell death and induces no or minor CPE (see above), various animal tumor models were next explored in our laboratory to test PV oncoselectivity under *in vivo* conditions. The most extensive animal studies were performed using two tumor models, namely glioma and pancreatic ductal adenocarcinoma (PDAC), which are distinguished by a dismal prognosis and represent potential targets for H-1PV-based oncolytic virotherapy clinical applications. Rats, i.e., the natural permissive host of the virus, were engrafted with the respective tumors, and a long-term follow-up of the animals was performed after H-1PV treatment, in order to assess the tumor-suppressing capacity of the virus, and to reveal any signs of virus-induced toxicity and/or other adverse effects.

### Glioma

Only about 50% of the patients with malignant brain tumors of glial origin survive 1 year after initial diagnosis. Furthermore, standard treatment options lead to only modest improvements in glioma outcome (Stummer et al., 2006). Oncolytic H-1PV, whose capacity to selectively kill glioma-derived cells through a cathepsin-mediated mechanism was demonstrated *in vitro* (Di Piazza et al., 2007), is believed to represent a promising therapeutic alternative. In this regard, experimental evidence obtained in our laboratory showing that intracerebral or systemic H-1PV injection leads to glioma regression in immunocompetent rats bearing orthotopic autologous RG-2 tumors and in immunodeficient animals implanted with human U87 gliomas without causing any toxic side effects, is of major preclinical importance (Di Piazza et al., 2007; Geletneky et al., 2010; Kiprianova et al., 2011). These effects could not only be achieved by direct local intratumoral therapy but also after intravenous and even intranasal virus inoculation, although significantly higher virus doses had to be used for systemic application. The effect of intracerebral H-1PV injection applied to healthy or glioma-bearing rats on CTSB activity was investigated by Di Piazza et al. (2007). In healthy brains, CTSB activity was found to be very low and not significantly affected by H-1PV treatment. In contrast, in H-1PV-treated gliomas a striking enhancement of CTSB activity was detected, together with an increase in the total amount of tumor-associated enzyme. Histological examination of H-1PV-treated gliomas raised in immunocompetent rats (Geletneky et al., 2010) demonstrated that, in addition to causing remission and improving animal survival, H-1PV treatment was not associated with normal brain tissue or other organs damage and was accompanied by only minor signs of inflammation. In agreement with previous *in vitro* and *in vivo* data (Di Piazza et al., 2007), CTSB activation was observed only in H-1PV-infected tumor cells but not in the surrounding peritumoral tissue. Parvoviral DNA could be detected in the tumor and in the peritumoral brain tissue 48 h post infection (p.i.). Virus spreading further increased with time and at 72 h p.i. viral DNA could additionally be detected in the contralateral brain hemisphere, in the cerebellum, and in distant organs (heart, lungs, liver, spleen, and kidneys), yet only transiently since no viral DNA could be revealed in any normal tissue 2 weeks p.i. Virus transcription and NS1 accumulation were strongly restricted to the tumor remnants, while undetectable in surrounding normal tissues, thus arguing for the selective H-1PV replication in tumor cells. To confirm this statement, virus replication was compared in two groups of animals, i.e., in glioma-bearing and in healthy control rats. The infectious virus yield in the brain of H-1PV-treated tumor-bearing rats was two orders of magnitude higher than in the brain of control animals, which were tumor-free but have been injected with the same amount of virus. This preferential replication of H-1PV in the neoplastic tissue, without inducing histopathological changes in normal brain tissue, provided convincing evidence that H-1PV preserves the oncotropism displayed in cell cultures under *in vivo* conditions as well (Geletneky et al., 2010).

In an immunodeficient rat model of human glioma, both intratumoral and multiple systemic (intravenous) H-1PV administrations were tested and shown to result in tumor suppression without being accompanied by any treatment-associated side effects. All animals subjected to H-1PV virotherapy remained active and gaining weight until the end of the observation period (Geletneky et al., 2010). Expression of the PV protein NS1 was detected in the necrotic tumor areas but not in the surrounding normal brain tissue, in agreement with the observations made in glioma-bearing immunocompetent animals (see above). The work of Geletneky et al. further raised the question whether an intranasal H-1PV application might also achieve therapeutic efficiency and suppress the growth of human glioma xenografts in immunodeficient rats. As reported by Kiprianova et al. (2011), a single intranasal virus instillation led to significant glioma regression and animal survival prolongation, without any toxicity for all but tumor tissues. Indeed, all virus replication markers were expressed exclusively in the tumor. These results suggest a safe and efficient alternative to H-1PV administration via the standard invasive intracranial route.

### Pancreatic ductal adenocarcinoma

Pancreatic ductal adenocarcinoma is one of the most lethal gastrointestinal malignancies, causing every sixth cancer-related death in Europe (Jemal et al., 2007). The disease is highly resistant to current treatments: surgical resection, which achieves the best long-term survival so far, is feasible in only a minority of patients (Finlayson and Birkmeyer, 2003). Oncolytic H-1PV infection of PDAC-derived cells *in vitro* was shown to result in efficient virus-induced cell death, even when the cells were resistant to standard chemotherapeutics, e.g., gemcitabine (Angelova et al., 2009b). Furthermore, H-1PV capacity to suppress PDAC was also studied *in vivo*. In a syngeneic orthotopic rat model of PDAC, a single intratumoral H-1PV injection was applied 2 weeks after implantation of rat pancreatic carcinoma cells into the pancreas. Virus expression assessment demonstrated that H-1PV was expressed selectively in tumor as opposed to normal tissues. An initial burst of virus expression in tumor and surrounding pancreatic tissue was observed shortly after virus injection. H-1PV transcripts were also detected in lymphoid organs. From day 10 onwards, virus expression faded in normal pancreatic and other distant visceral tissues but remained persisting in the tumor (Angelova et al., 2009b). In an immunodeficient environment, a similar selective tumor targeting and absence of toxicity were observed in PDAC-bearing H-1PV-treated nude rats (Li et al., 2013).

In another study, *human cervical carcinoma* xenograft-bearing nude rats were used to demonstrate a virus dose-dependent tumor growth arrest and regression. NS1 expression was detected only in the kidneys and at very low levels. Remarkably, no weight loss or other adverse effects were documented in any of the treated animals (Li et al., 2013).

Recently, two large-scale animal experiments using virus-permissive immunocompetent rats were initiated in order to provide further preclinical proofs of H-1PV favorable safety profile. The virus was applied intravenously to healthy rats as either a single high dose or multiple injections. Virus doses were considered equivalent or higher than those resulting from viral amplification after infection of brain tumors. It is known that the rat is the natural host of H-1PV and the latter may persist in normal rat populations by a mechanism not yet known. This persistence indicates that also non-tumor-bearing rats are capable for parvovirus replication, making this animal model a suitable choice for the detection of possible side effects of H-1PV therapeutic application. Irrespective of the administration regimen used, animal mortality or macroscopic organ changes were not observed. Minimal diffuse bile tract hyperplasia and germinal center development in the spleen were detected after multiple H-1PV applications. However, liver changes were reversible within a 2-week recovery period. No virus-induced toxic effects could be revealed by measuring blood parameters (hematology, chemistry, coagulation). In agreement with *in vitro* data, blood mononuclear cells showed no functional alterations after virus injection, and measurable cytokine release could be detected. H-1PV treatment led to the development of IgG antibodies. The virus was shed mainly via feces (Geletneky et al., 2015a). Furthermore, the same authors demonstrated that H-1PV is non-pathogenic in adult rats and infection does not affect central or autonomous nervous system functions, even after a direct injection into the brain (Geletneky et al., 2015b).

Taken together, the above described data provide experimental evidence of the oncoselectivity of H-1PV in adult animals from its natural host, the rat, resulting in tumor suppression in absence of any pathological signs (Table 2). This innocuousness was also shown in young rats (Jacoby and Ball-Goodrich, 1995; Gaertner et al., 1996), in agreement with previous studies showing that pathological effects could only be observed if infection of the animals took place within the first few days after birth (Jacoby et al., 1979). Overall, this favorable safety profile supports the further translation of H-1PV applications into the clinic.

**Table 2:** ***In vivo* evidence of H-1PV oncoselectivity**.

Tumor (Tu)	H-1PV adm. route	Normal tissue (N)^a^	Tu versus N sensitization to H-1PV infection	Reference
Rat/human pancreatic carcinoma	i.t.	Normal pancreatic and other visceral tissues	Long-lasting H-1PV expression in Tu Transient H-1PV expression in N; no virus-induced changes in blood, liver, and kidney clinical parameters	Angelova et al. (2009b) and Li et al. (2013)
Rat/human glioma	i.t.	Normal brain and other visceral tissues	Late H-1PV expression in residual Tu; Tu-dependent H-1PV production in brain	Di Piazza et al. (2007), Geletneky et al. (2010), and Kiprianova et al. (2011)
	i.v.		
	i.n.		Transient virus genome detection, no late virus expression and no pathological alterations in N	
Human cervical carcinoma	i.t.	Normal visceral tissues	Selective NS1 expression in Tu No weight loss or other side effects	Li et al. (2013)
Immunocomp. Tu-free rats	i.v.	Visceral tissues	Broad organ distribution and time-dependent decrease of H-1PV genomes	Geletneky et al. (2015a,b)
	i.c.		
			No or minimal and reversible toxicological changes

*^a^Treated animals were immunocompetent (no tumor or rat tumor grafts) or nude (human tumor xenografts) *rats**.

*i.t., intratumoral; i.v., intravenous; i.n., intranasal; i.c., intracerebral*.

## H-1PV Anti-Tumor Efficacy Assessment Using Xenografts Models in Non-Permissive Mice

Additional studies (summarized in Table 3) were carried out to test the suppressive activity of H-1PV on human tumor xenografts in immunodeficient recipient mice. Since the mouse is a non-permissive host for H-1PV, these studies are not informative regarding the oncoselectivity of H-1PV, but are much relevant for the assessment of the suppressing capacity of the virus against human tumor targets.

**Table 3:** **H-1PV-induced suppression of human tumor xenografts in mouse models**.

Tumor entity (tumor model)	H-1PV-induced anti-tumor effects	Reference
Breast carcinoma (HMEC HBL100 cells s.c. implanted in nude mice)	Tumor growth suppression and complete remission with no recurrence in 50% of the virus-treated animals	Dupressoir et al. (1989)
Cervical carcinoma (HeLa cells s.c. implanted in SCID mice)	Complete tumor regression after high virus dose application	Faisst et al. (1998)
Burkitt lymphoma (Namalwa cells s.c. implanted in SCID mice)	Efficient tumor regression and necrosis. Virus-induced effects also after application at late disease stages	Angelova et al. (2009a)
Gastric carcinoma (SGC-7901 cells or MKN28, SGC7901, MKN45 cells transfected with NS1-expressing plasmid, s.c. implanted in nude mice)	Tumor growth suppression by *in vivo* virus infection or *ex vivo* NS1 transduction	Zhang et al. (1997) and Wang et al. (2012)
Pancreatic carcinoma^a^ (human BxPC-3 cells s.c. implanted in nude mice; PDAC operational material s.c. implanted in SCID mice)	H-1PV dose-dependent tumor growth delay	Angelova et al. (2009b) and Li et al. (2013)

*^a^Implantation of non-established (patient-derived) tumor material*.

*HMEC, human mammary epithelial cells; s.c., subcutaneously; PDAC, pancreatic ductal adenocarcinoma*.

H-1PV capacity to inhibit the growth of human tumors xenotransplanted in recipient immunocompromised mice was first documented by Dupressoir et al. (1989) and further substantiated by Faisst et al. (1998). Dupressoir et al. reported that both local (intratumoral) and systemic (intravenous) virus application lead to significant *human mammary carcinoma* growth suppression and, in some cases, reversion, without any detectable infection-associated deleterious side effects. In the work of Faisst et al., quickly growing subcutaneous carcinomas were established by implantation of *human cervical carcinoma* cells into immunodeficient SCID mice. Tumor-bearing animals were subsequently intratumorally injected with different H-1PV doses. Intratumoral H-1PV gene expression and dose-dependent tumor regression were observed. This study provided the experimental evidence showing that a single local H-1PV injection is sufficient to induce regression, in an immunodeficient recipient environment, of certain human solid carcinomas.

Similar observations were made by Angelova et al. (2009a) in a human lymphoma model. A single intratumoral H-1PV injection in *human Burkitt lymphoma*-bearing SCID mice resulted in quick tumor regression. Moreover, it was shown that low H-1PV doses can achieve a strong therapeutic effect as well, even when the virus was applied at advanced disease stages. Histochemical analyses demonstrated H-1PV spreading to distant, non-virus-treated tumors, in addition to intratumoral replication and protein expression in virus-treated tumor tissues.

Wang et al. (2012) reported that in an *in vivo human gastric carcinoma* model the *ex vivo*-induced NS1 expression in poorly differentiated gastric cancer cells prevents them from forming tumors in nude mice. This study was in agreement with an earlier report (Zhang et al., 1997) showing that intratumoral H-1PV application to human gastric carcinoma-bearing mice results in efficient tumor inhibition. Importantly, this oncosuppression was not accompanied by any toxic side reactions, even when the virus was applied as long as twice per week for 3 weeks. Similar lack of toxicity was observed after an intraperitoneal animal treatment.

Tumor regression and complete remission in *human pancreatic carcinoma*-bearing nude mice (Angelova et al., 2009b) or NOD/SCID mice (Li et al., 2013) were observed after the critical H-1PV dose was reached in a virus dose-escalation experiment. This regression was not associated with detectable toxicity. Importantly, pancreatic carcinoma patient-derived primary tumor material was used in the study by Li et al. (2013).

## Preclinical Testing of H-1PV-Based Combinatorial Treatments

In order to evaluate the cooperative capacity of *H-1PV and gemcitabine* for suppressing pancreatic carcinoma growth in an orthotopic rat model, tumor-bearing animals were sequentially treated first with gemcitabine and 2 weeks later with H-1PV in a two-step protocol (Table 4). Since the two agents induce different death pathways (apoptosis and cathepsin-mediated cell death, respectively), H-1PV-triggered oncolysis is expected to circumvent the antiapoptotic features (i.e., gemcitabine resistance) acquired by many tumor cells during PDAC progression. The data have indeed demonstrated that the anti-PDAC potential of the drug was significantly improved when parvovirus was added to the treatment. The combined treatment was not accompanied by additive toxicity, as illustrated by the results of an extensive toxicological assessment carried out in this model (Angelova et al., 2009b). Rat bone marrow, liver, and kidney functions were monitored by measuring clinically relevant makers. The blood-borne markers of bone marrow activity were unaffected, except for the gemcitabine-induced drop in the reticulocyte and monocyte levels. Bilirubin, aspartate aminotransferase (ASAT), and alanine aminotransferase (ALAT) levels were elevated in both untreated and gemcitabine-treated groups of animals, reflecting the low-grade lytic processes typical of the livers of PDAC-bearing rats. H-1PV application as a second-line treatment restored the levels of these markers to their respective normal physiological ranges. Creatinine levels remained stable after the combinatorial treatment, showing an unaltered kidney clearance. It was concluded from the above toxicological assessments that the blood-borne marker variations detected were fully attributable to gemcitabine treatment and were not aggravated by subsequent H-1PV administration.

**Table 4:** **Safety of H-1PV-based combinatorial treatments**.

Tumor entity	H-1PV combination partner	Cooperative anti-neoplastic effects	Oncoselectivity	Reference
Pancreatic carcinoma	Gemcitabine	Enhanced effectiveness of combinative treatment in *in vivo* tumor suppression	No H-1PV-induced changes in blood-borne bone marrow activity	Angelova et al. (2009b)
			Unaffected kidney and liver functions	
	VPA^a^	Effective H-1PV dose *in vivo* much reduced when VPA added to treatment	Selective tumor targeting No weight loss	Li et al. (2013)
Cervical carcinoma	VPA	Synergistic tumor growth arrest *in vivo*	No other signs of toxicity	

*^a^Implantation of non-established (patient-derived) tumor material*.

*VPA, valproic acid*.

Similar observations were made when established PDAC or cervical carcinoma cells, as well as primary PDAC patient-derived tumor material, were used for implantation into immunodeficient rats or NOD/SCID mice, respectively (Li et al., 2013), in order to assess the anti-tumor efficacy and biosafety of H-1PV combined with valproic acid (VPA). VPA belongs to the class of histone deacetylase (HDAC) inhibitors and has been shown to reinforce the cytotoxicity of many OVs, including the vesicular stomatitis virus (Alvarez-Breckenridge et al., 2012), herpes simplex virus (Otsuki et al., 2008), and adenoviruses (Van Oosten et al., 2007), by inhibiting the expression of cellular genes involved in antiviral immune responses and/or by stimulating the expression of genes required for the viral life-cycle (Nguyen et al., 2010). *In vitro*, VPA has been shown to enhance H-1PV NS1-mediated cytotoxicity through increased protein acetylation and upregulated transcriptional activity (Li et al., 2013). In agreement with *in vitro* data, H-1PV treatment *in vivo* followed by VPA administration, was devoid of weight loss or other signs of toxicity, and resulted in synergistic tumor regression and survival prolongation. In addition, this combinatorial approach allowed reduction of H-1PV doses to a level, which is suboptimal in a monotherapeutical setting.

**Figure 4:**
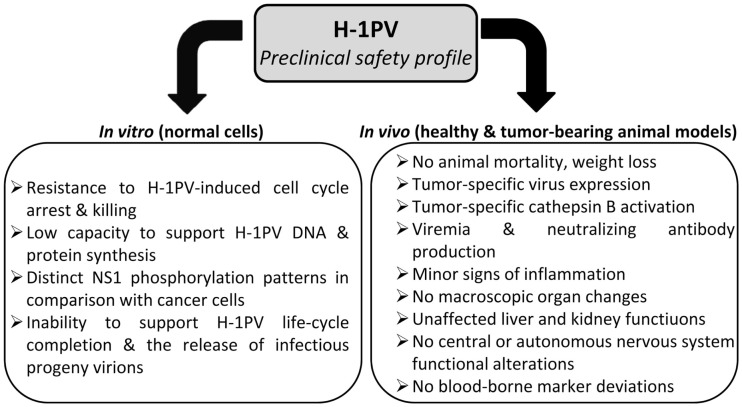
**Preclinical evidence of H-1PV safety**.

Altogether, preclinical data convincingly argue against a significant risk of oncolytic parvovirus H-1 inducing severe toxic effects when applied to the human patient. Moreover, H-1PV treatment is unlikely to pose a risk of insertional mutagenesis since autonomous parvoviruses are not known to integrate into the host cell genome (Richards and Armentrout, 1979; Ron and Tal, 1985). This highly favorable safety profile (Figure 4) together with prominent anti-cancer activities justify H-1PV consideration as mono-, combined, or second-line treatment alternative to current conventional toxic strategies.

## Clinical Evidence of H-1PV Tolerability

Despite frequent H-1PV isolation from cancer patients’ tissues and transplantable tumors or as a contaminant of human tumor-derived cell lines in 1970s (Toolan et al., 1960), all attempts to isolate the virus from normal non-malignant human tissues failed (Toolan et al., 1962; Rommelaere and Tattersall, 1989). No convincing experimental evidence of an association between any human disease and a previous H-1PV infection could be found either. Initial indications of a possible role of H-1PV in gestational problems were not confirmed by subsequent studies, which failed to reveal the presence of H-1PV-specific antibodies or H-1PV virions in sera or tissue specimens obtained after spontaneous human abortions (Monif et al., 1965; Neuman et al., 1970).

Important early clinical evidence showing that H-1PV application in humans is well tolerated and devoid of side toxicity was provided after experimental infection of human cancer patients under a compassionate use agreement. In the pioneering study by Toolan et al. (1965), two patients (a 12- and 13-year-old girl) with advanced disseminated osteosarcoma were intramuscularly inoculated with H-1PV at a dose of approximately 1 × 10^9^ plaque-forming units (pfu) of a non-GMP-virus formulation. In one of the patients, a direct virus injection into the tumor of the right hip area was also applied. One of the patients developed fever of up to 38.5°C within the first 10 days after virus administration, yet it was not clearly attributable to the virus inoculation because of the presence of a concomitant urinary tract infection. Body temperature returned to normal after 5 days. The patient was discharged at day 15 without any additional clinical symptoms but she died of tumor progression shortly after hospital readmission at day 38 after H-1PV treatment. The second patient suffering from advanced metastatic disease did not experience any virus treatment-associated side effects. In both patients, H-1PV injection led to an extensive viremia and production of H-1PV-specific neutralizing antibodies. Overall, no virus-induced organ-specific side effects could be observed and H-1PV safety and tolerability was considered as good in both patients.

In a later study, which took place in France within the frame of a clinical trial entitled “Phase I clinical study on possible use of H-1 parvovirus in cancer treatment,” 12 patients with skin metastases originating from different types of solid tumors (breast adenocarcinoma, melanoma, lung large cell carcinoma, pancreatic carcinoma, and kidney leiomyosarcoma) were subjected to an intralesional dose-escalation (1 × 10^8^, 1 × 10^9^, 1 × 10^10^ pfu) H-1PV treatment (Le Cesne et al., 1993). The virus was administered repeatedly, with 10-day treatment-free intervals. Seroconversion was detected at days 10–15 after the first virus injection. Only a moderate fever up to approximately 38°C (in 3 out of the 12 patients), an isolated increase of creatinine and gamma-glutamyl transferase (GGT) but no other H-1PV-associated toxic side effects could be observed, arguing for an excellent safety profile of this oncolytic virus in humans. It is also noteworthy that in two out of seven breast carcinoma patients stable disease was documented throughout the observation period. The presence of virus DNA/proteins in tumor extracts was investigated in four subjects and in all of them viral genomes/proteins could be found after administration of H-1PV in both target lesions (metastases to which the virus was administered) and in control lesions (metastases distant from the site of injection). This confirmed a systemic exposure to the virus as already demonstrated by viremia.

With the support from this encouraging safety data in humans, a next clinical trial with H-1PV in patients with malignant brain tumors was planned and initiated (Geletneky et al., 2012, 2014a,b). This trial used three modes of virus application that were not tested in 1965 and 1993 reports, namely intratumoral injection, injection directly into the brain parenchyma bordering the tumor, and intravenous injections. From the aspect of safety and tolerability, these routes of parvovirus administration were potentially more challenging since active virus particles in the central nervous system (CNS) could lead to encephalitis or meningitis, and an intravenous infusion might result in a more rapid systemic exposure compared to the H-1PV release into the circulation after intramuscular, subcutaneous, or intracutaneous injection. The trial was planned as a dose-escalation trial at dose ranges of 1 × 10^6^, 5 × 10^7^, and 1 × 10^9^ pfu. The H-1PV preparation was produced according to GMP standards thereby providing certified virus concentrations and purity.

The initial treatment was by intratumoral injection of half of the total dose per patient followed by a 9-day observation period in which the virus could interact with the tumor. Tumor resection was performed at day 10 and the second half of the dose was given by multiple injections into the tumor-surrounding brain. Three patients per dose group were treated and, for safety reasons, a time interval of 28 days (between the first and the second patient) or of 18 days (between the second and the third patient of one group) was allowed. All virus injections were well tolerated without any virus-associated side effects or pathology. Some adverse effects were detected but they were rated unrelated to virus treatment and no dose-limiting toxicity has been found. We did not detect any signs of fever or flu-like symptoms and in particular no signs of CNS pathology. Measurements of viral genomes in blood showed positive results in the intermediate-dose and high-dose group indicating penetration of H-1PV through the blood–brain barrier, again with no signs of systemic toxicity.

After the safety data of the intratumoral patient arm had been reviewed by external experts and by the responsible federal agency (Paul-Ehrlich Institut, Langen, Germany), the permission was granted to move on to the intravenous trial arm (Geletneky et al., 2014a,b). Here, the patients were injected with a total dose of 5 × 10^7^ or 1 × 10^9^ pfu of H-1PV. All patients received daily virus infusions of 10% of the total dose at days 1 through 5, followed by a 4-day recovery period. At day 10, surgery and intraparenchymal injection of the second half of the planned dose of H-1PV were performed, as in the first trial arm. Also, after intravenous injection, we did not observe any side effects, in particular, no fever and no signs of typical virus infections. The pharmacokinetic measurements showed measurable concentrations of H-1PV over the first 5 days (and in some patients for another one), and virus DNA was constantly present in the 22 h period between the daily injections indicating continuous exposure.

Another important aspect of the safety of H-1PV treatment is biosafety and possible virus shedding by treated patients. Therefore, patients were hospitalized under quarantine conditions until they had generated an antibody response or were found to be negative for H-1PV in shedding samples (feces, urine, and saliva). Only small amounts of virus DNA could be detected in some feces probes while saliva and urine were constantly negative. It is still unknown whether the positive test results are indicative of active and infectious virus or of virus nucleic acids only.

In conclusion, based on three applications in humans, H-1PV can be considered safe and well tolerated at least to a cumulative dose of 1 × 10^9^ pfu. Flu-like symptoms can occur under treatment but this could not be confirmed in the last trial with GMP-virus. Thus, it cannot be ruled out that these symptoms were related to impurities of the virus preparation and not caused by the virus itself. Another consequence of the current safety data is that the need for patients to be kept under isolation should be reconsidered for future trials.

## Conflict of Interest Statement

Jean Rommelaere received research grants from ORYX GmbH & Co.KG. Assia L. Angelova, Karsten Geletneky, and Jean Rommelaere have an ownership interest (including patents) in the German Cancer Research Center. Jürg P. F. Nüesch has no conflicts of interest to declare.
